# Atopic dermatitis is associated with herpes zoster in adults – A matched cohort study using electronic health records data in England

**DOI:** 10.1111/ddg.70329

**Published:** 2026-08-05

**Authors:** Anna K. Schober, Sinéad M. Langan, Hywel C. Williams, Harriet Forbes, Julian Matthewman

**Affiliations:** ^1^ Department of Non‐Communicable Disease Epidemiology London School of Hygiene and Tropical Medicine United Kingdom of Great Britain and Northern Ireland; ^2^ Centre of Evidence Based Dermatology Lifespan and Population Health, School of Medicine, University of Nottingham, Nottingham, United Kingdom of Great Britain and Northern Ireland

**Keywords:** Atopic dermatitis, atopic eczema, cohort study, eczema, electronic health records, herpes zoster, shingles

## Abstract

**Background and objectives:**

Some atopic dermatitis (AD) treatments have been linked to an increased risk of herpes zoster (HZ). Limited evidence suggests an independent association between atopic dermatitis and herpes zoster. The study aimed to investigate the association between atopic dermatitis and herpes zoster in the UK and to explore the role of treatments in this relationship.

**Patients and methods:**

We conducted a matched cohort study using routinely collected primary care data from the UK Clinical Practice Research Datalink (CPRD) Aurum database (1997–2023) and applied Cox regression models. Age, behavioral factors, several comorbid diseases and different treatment exposures were included as covariates.

**Results:**

Individuals with atopic dermatitis had an adjusted hazard ratio of 1.28 (95% CI: 1.27–1.29) for HZ. Adjustments for different definitions of oral corticosteroid exposure as well as other traditional immunosuppressants led to minimal reductions in the association. The risk of herpes zoster increased with AD severity (aHR for mild AD: 1.22 [95% CI: 1.20–1.23]; aHR for moderate AD: 1.28 [95% CI: 1.27–1.30]; aHR for severe AD: 2.33 [95% CI: 2.22–2.45]).

**Conclusions:**

The risk of HZ is increased in individuals with atopic dermatitis, independent of comorbidities and the use of oral corticosteroids or immunosuppressants. This elevated baseline risk is particularly relevant because herpes zoster is also a known adverse effect of newer therapies such as Janus kinase (JAK) inhibitors. These findings may inform vaccination guidelines.

## INTRODUCTION

Atopic dermatitis (AD), also known as atopic eczema,^1^ is a chronic inflammatory skin disease imposing a significant burden on patients and healthcare systems in terms of disability‐adjusted life‐years.^2^, ^3^ While prevalence is highest in children, there is also a substantial disease burden in adults, with estimated prevalences of around 4.4% for adults in Europe, ranging from 2.2% in Germany and 4.3% in the UK, to 8.1% in Italy.^4^, ^5^, ^6^ The disease can manifest both chronically or relapsing‐remitting and is characterized by intense itching and eczematous lesions.^7^

People with severe AD may require systemic treatment due to recalcitrant disease.^8^ This may involve oral corticosteroids for acute flares and immunosuppressants like ciclosporin or methotrexate for ongoing management. In recent years, several biologic and small‐molecule treatments have been developed and approved as further promising treatment approaches.^9^

Previous research on AD has found a range of associations with other comorbidities, including an increased disposition towards systemic infectious diseases.^10^, ^11^ A recent study^12^ found skin infections, including herpes simplex, to be amongst the adverse outcomes most strongly associated with AD, but did not investigate its association with herpes zoster.

Herpes zoster (HZ), commonly known as shingles, results from the reactivation of latent varicella‐zoster virus (VZV) in cutaneous nerves, particularly in older adults and immunosuppressed individuals.^13^, ^14^ It presents with a distinct painful, unilateral rash in the corresponding dermatome.^15^

The potential association between AD and HZ has predominantly been discussed in the context of AD treatments. In particular, systemic corticosteroids and immunosuppressants have been associated with an increased risk of HZ,^16^, ^17^ as have JAK inhibitors (JAKi), a subclass of newly developed small‐molecule therapies.^8^ However, due to the altered skin immune response of people with AD and their previously observed increased susceptibility to both cutaneous and extracutaneous infections,^10^, ^18^ it is plausible that people with AD might have an elevated risk of herpes zoster independent of their treatment.

There is some evidence suggesting an increased risk of HZ in individuals with AD independent of systemic treatments,^19^, ^20^, ^21^, ^22^ but the overall evidence regarding the strength, magnitude, and generalizability of the association between AD and HZ remains limited. A large UK cohort study has focused on the “VZV infection risk” without specifically looking at chicken pox or HZ diagnoses.^19^ Other studies lack precision,^20^, ^21^ or are based on hospital data^22^ and therefore not generalizable to the general population.

Considering vaccine developments and a broader roll‐out of vaccination and prevention schemes, further and more precise investigation of the possible association can help adequately inform vaccination guidelines and stratified risk assessments for people with AD.

The present study aims to investigate the relationship between AD and HZ among adults using UK primary care data, while considering the potential complex role of systemic immune modifying treatments and oral corticosteroid exposure.

## PATIENTS AND METHODS

This study is a matched cohort study utilizing routinely collected primary care data from the UK's Clinical Practice Research Datalink (CPRD) Aurum. The CPRD Aurum represents a large research database containing over 46 million routinely collected and de‐identified primary care electronic health records (EHR) in England. It has been found to be representative of the English population regarding geographical distribution, deprivation level, gender and age.^23^

People with AD in the database were identified based on a previously validated algorithm that considered a combination of a corresponding diagnostic code and at least two AD‐related prescriptions.24 The index date (i.e., the date of cohort entry) was defined as the latest of the following:

– the date on which the AD definition was met;

– one year after registration with a general practitioner (GP);

– the study start date (April 1, 1997); or

– the date on which the individual turned 18 years of age.

This resulted in a cohort including both incident and prevalent cases.^25^ People in the AD cohort were then matched on age (± 2 years), sex, and general practice to up to five unexposed comparators, i.e. individuals who did not meet the AD definition. Comparators were followed up from the same date as the corresponding exposed individual.

If comparators met the AD definition during follow‐up, they were censored and subsequently re‐matched as exposed patients. All individuals were otherwise censored at the first of the following dates:

 diagnosis of the study outcome (HZ);

– date of leaving the GP practice;

– death; or

– study end (March 31, 2023).

Observations were excluded from the study if any diagnosis of HZ was documented in their records prior to their index date. The analysis was restricted to matched sets that contained both exposed and unexposed individuals. The outcome of interest was the first documented diagnosis of HZ (See code list in Supplement S1).

A range of covariates was considered and included in the analysis as potential confounders. This included a range of chronic comorbidities such as asthma,^19^, ^26^, ^27^ allergic rhinitis,^19^, ^26^, ^28^ COPD,^19^, ^28^, ^29^ coronary artery disease,^28^, ^30^ Crohn's disease,^19^, ^31^, ^32^ depression,^19^, ^28^, ^33^ diabetes,^19^, ^28^, ^34^, ^35^ heart failure,^19^ hypertension,^28^, ^34^ and ulcerative colitis.^19^, ^31^, ^32^ A documentation of these comorbidities prior to a patient's index date was considered as positive history of the disease. A diagnosis of obesity, smoking status, or alcohol abuse within the 2 years preceding the index date was considered positive for these covariates. Quintiles of Index of multiple deprivation (IMD) scores were extracted from the IMD database to assess social deprivation.^36^ Information on ethnicity was extracted from the CPRD Aurum observation data.^37^, ^38^

We used CPRD Aurum prescription data to define drug exposure status. Binary time‐updated variables were created for ever‐exposure to oral corticosteroids and/or systemic immunosuppressants (methotrexate, azathioprine, ciclosporin, or mycophenolate), defined as first exposure occurring within 180 days before the index date or during follow‐up. To further explore the effects of oral corticosteroids, we additionally considered prescriptions in the year before the index date and the recency of the last prescription (< 30 days, 30–90 days, 90–180 days, and > 180 days) as time‐updated variables.

AD severity was approximated based on treatment patterns: patients were classified as having moderate AD after receiving prescriptions for either potent topical corticosteroids or calcineurin inhibitors, and as having severe AD after receiving systemic immunosuppressants or having a record of phototherapy. They were assumed to have mild AD otherwise. The assigned severity could progress to higher levels but not revert to a lower level. This aligns with previous studies. Due to limited data availability, information from hospital records was not included.^12^ All utilized code lists can be found in the repository of a previous study.^12^

Stratified Cox proportional hazards regression models were used to calculate hazard ratios (HRs) and corresponding 95% confidence intervals (CIs). The matching variables were implicitly accounted for. Calendar period of follow‐up was addressed through matched index dates. A univariable stratified model was initially fitted to investigate the association while accounting only for matching factors and was subsequently extended to a multivariable model including the remaining covariates. We then added the different definitions for treatment exposures to assess their role in the relationship. To investigate the influence of age on effect estimates, observations were split into 10‐year age bands and an interaction term between AD and age was included. Schoenfeld residuals were assessed to evaluate time‐dependence. Due to high levels of missingness, IMD scores (13.6% missing) and ethnicity (24.6% missing) were not included as covariates in the main statistical analysis.

Sensitivity analyses were undertaken to explore how different inclusion criteria affected the effect estimates; this included restricting the cohort to incident AD cases (where the index date matched the AD diagnosis date) and removing the age restriction to include children. A further sensitivity analysis was performed by restricting observations to individuals with at least one of four common records in the year before the index date (9N11.00 – Seen in GP's surgery; 22A¨00 – Body weight; 4….00 – Laboratory test; 246¨00 – O/E – blood pressure reading). We additionally adjusted for ethnicity and the IMD.

## RESULTS

### Baseline characteristics

A total of 14,081,774 observations from 13,426,267 people were included in the study: 11,636,684 observations from AD‐unexposed participants and 2,445,090 from AD‐exposed participants (1,382,577 incident cases and 1,062,513 prevalent cases). In total, 84,341,513 person‐years at risk (15,729,178 exposed years and 68,612,336 unexposed years) were observed. A total of 329,821 HZ events occurred: 76,990 in people with AD and 252,831 in the matched comparator group. A comprehensive overview of participant characteristics is shown in Table 1.

**TABLE 1:** Baseline characteristics of patients (n, %) stratified by AD exposure status. Patients could contribute person‐time to both groups; therefore, the sum of observations does not reflect the total number of participants.

	No AD (n = 11,636,684)		AD (n = 2,445,090)	
	*n*	*%*	*n*	*%*
Zoster events	252,831	(2.2%)	76,990	(3.1%)
Age (mean [SD])	40.7	(20.5)	41.4	(20.9)
Sex				
F	6,750,742	(58.0%)	1,422,616	(58.2%)
M	4,885,934	(42.0%)	1,022,466	(41.8%)
I	8	(< 0.1%)	8	(< 0.1%)
IMD^2^				
1 (least deprived)	2,033,670	(17.5%)	435,207	(17.8%)
2	2,041,860	(17.5%)	430,571	(17.6%)
3	1,984,581	(17.1%)	411,505	(16.8%)
4	2,054,895	(17.7%)	427,836	(17.5%)
5 (most deprived)	1,935,259	(16.6%)	404,905	(16.6%)
missing	1,586,419	(13.6%)	335,066	(13.7%)
Ethnicity				
White	6,024,957	(51.8%)	1,301,908	(53.2%)
South Asian	554,860	(4.8%)	138,197	(5.7%)
Black	279,368	(2.4%)	55,606	(2.3%)
Other	1,496,796	(12.9%)	365,280	(14.9%)
Mixed	91,613	(0.8%)	17,853	(0.7%)
Not stated	278,563	(2.4%)	51,873	(2.1%)
Missing	2,910,527	(25.0%)	514,373	(21.0%)
Alcohol abuse	42,954	(0.4%)	13,434	(0.5%)
Cigarette Smoking	2,121,192	(18.2%)	519,926	(21.3%)
Obesity	439,161	(3.8%)	137,096	(5.6%)
***History of***				
Asthma	1,485,154	(12.8%)	602,427	(24.6%)
Allergic rhinitis	836,455	(7.2%)	358,844	(14.7%)
Heart disease	660,471	(5.7%)	182,288	(7.5%)
COPD	180,763	(1.6%)	58,171	(2.4%)
Crohn's disease	23,439	(0.2%)	8,976	(0.4%)
Depression	1,497,428	(12.9%)	440,430	(18.0%)
Diabetes	489,087	(4.2%)	129,717	(5.3%)
Heart failure	113,011	(1.0%)	32,526	(1.3%)
Hypertension	1,414,772	(12.2%)	361,940	(14.8%)
Ulcerative colitis	39,823	(0.3%)	13,517	(0.6%)

*Abbr*.: AD, atopic dermatitis; COPD, chronic obstructive pulmonary disease; HZ, herpes zoster; IMD, Index of Multiple Deprivation; SD, standard deviation

^1^ p‐values were derived from χ^2^ tests for categorical variables and t‐tests for continuous variables.

^2^ Quintiles of the Index of Multiple Deprivation.

### Incidence rates

The overall crude incidence rate of HZ in the entire cohort was 3.91 (95% CI: 3.90–3.92) per 1,000 person‐years. Crude incidence rates of HZ increased consistently with age, from 1.58 events per 1,000 person‐years among individuals aged < 30 years to 8.85 events per 1,000 person‐years among those aged ≥ 80 years. In the unexposed group, the rate was slightly lower with 3.68 events per 1,000 person years (95% CI: 3.67–3.70) than in the exposed group with 4.90 (95% CI: 4.86–4.93) events per 1,000 person years.

### Regression models

In the stratified model accounting for matching factors, individuals with AD had a 1.33‐fold higher hazard of HZ than their matched comparators without AD (95% CI: 1.31–1.34) (Table 2).

**TABLE 2:** Overview of results from univariable and multivariable Cox regression models: hazard ratios and 95% CIs.

	**HR**	**95% CI**
***Univariable model*** ^1^		
AD	1.33	(1.31–1.34)
***Multivariable model*** ^1^, ^2^		
AD	1.28	(1.27–1.29)
***Multivariable model*** ^1^, ^2^ ***+ different definitions of oral corticosteroid exposure ***		
Oral corticosteroid at baseline^3^		
AD	1.27	(1.26–1.28)
Oral corticosteroids (time‐updated)		
AD	1.24	(1.23–1.25)
Oral corticosteroid recency (time‐updated^4^)		
AD	1.25	(1.24–1.26)
Corticosteroids (recency)		
< 30 days	2.00	(1.95–2.05)
30–90 days	1.62	(1.57–1.67)
90–180 days	1.47	(1.42–1.52)
> 180 days	1.27	(1.26–1.29)
***Multivariable model*** 1, 2 ***+ immunosuppressants (time‐updated)***		
AD	1.27	(1.26–1.28)
Systemic immunosuppressants	2.06	(2.01–2.12)

*Abbr*.: AD, atopic dermatitis; CI, confidence interval; HR, hazard ratio

^1^ Implicitly adjusted for age, sex, general practice, and calendar period through matching on index date.

^2^ Adjusted for asthma, allergic rhinitis, COPD, coronary artery disease, Crohn's disease, depression, diabetes, heart failure, hypertension, ulcerative colitis, age at baseline, obesity, cigarette smoking, and alcohol abuse.

^3^ Record of corticosteroid prescription in the year prior to the index date.

^4^ Time since the last recorded corticosteroid prescription.

The model was then additionally adjusted for asthma, allergic rhinitis, COPD, coronary artery disease, hypertension, heart failure, ulcerative colitis, Crohn's disease, diabetes, depression, alcohol abuse, cigarette smoking, and obesity. These adjustments slightly decreased the effect estimate to an adjusted HR (aHR) of 1.28 (95% CI: 1.27–1.29).

The risk of HZ increased with increasing AD severity compared with individuals without AD (mild AD: aHR 1.22 [95% CI: 1.20–1.23]; moderate AD: aHR 1.28 [95% CI: 1.27–1.30]; severe AD: aHR 2.33 [95% CI: 2.22–2.45]).

### Effect of treatments

During follow‐up, 21.1% of patients with AD were prescribed oral corticosteroids compared with 9.7% of patients without AD. Potent topical corticosteroids were prescribed to 49.8% of individuals with AD compared with 10.0% of individuals without AD, while systemic immunosuppressants were prescribed to 1.9% of individuals with AD compared with 0.9% of individuals without AD.

Adjusting for oral corticosteroid use resulted in only minor attenuation of the adjusted effect estimates (Table 2). This was observed when oral corticosteroid exposure was assessed before the index date (aHR: 1.27; 95% CI: 1.26–1.28), as a time‐dependent covariate (aHR: 1.24; 95% CI: 1.23–1.25), or according to time‐updated prescription recency (aHR: 1.25; 95% CI: 1.24–1.26). Individually adjusting for time‐updated systemic immunosuppressants resulted in an aHR of 1.27 (95% CI: 1.26–1.28), whereas adjusting for topical corticosteroids resulted in an aHR of 1.17 (95% CI: 1.16–1.18).

### Interaction with age

**FIGURE 1:**
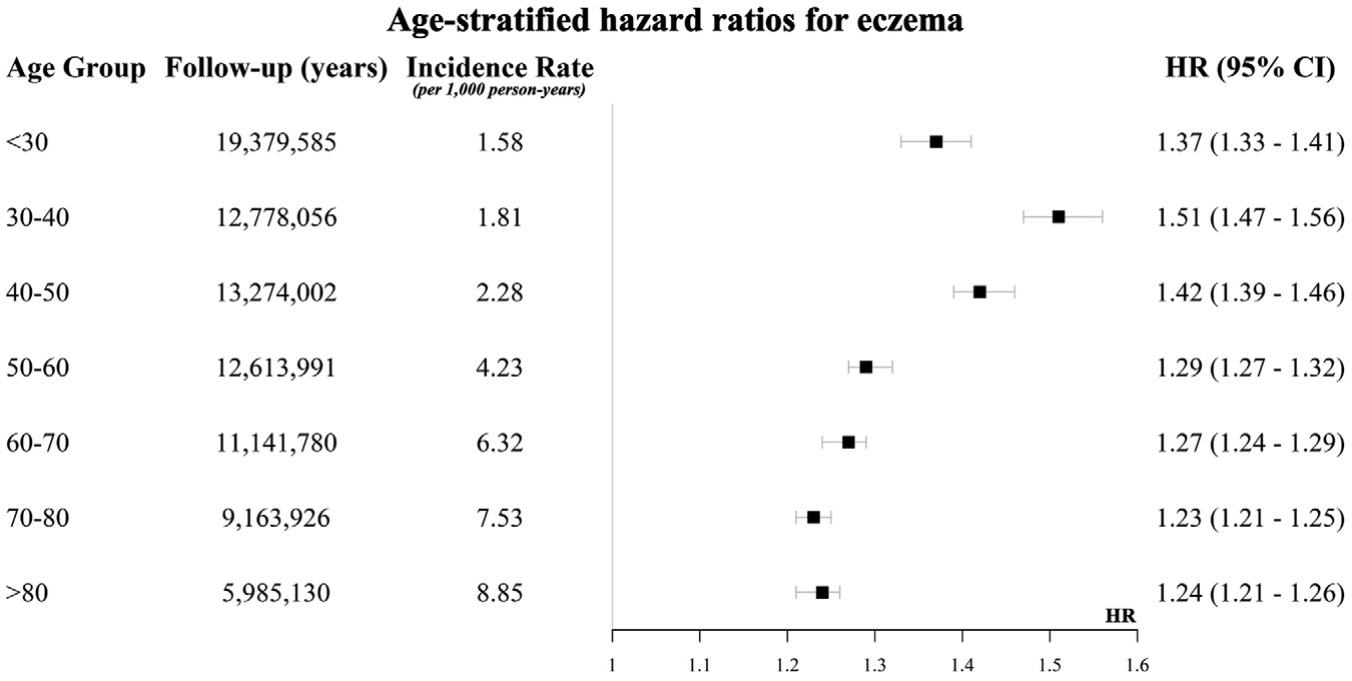
Forest plot showing hazard ratios (HRs) and 95% CIs for the association between HZ and herpes zoster stratified by age group, including an overview of follow‐up time and incidence rates for each group.

There was evidence of effect modification by age in the association between AD and HZ (likelihood ratio test: p < 0.001), with higher hazard ratios for AD observed in individuals younger than 50 years compared with older age groups (Figure 1, Table 3). Hazard ratios were highest among individuals aged 30–40 years (HR: 1.51; 95% CI: 1.47–1.56), followed by those aged 40–50 years (HR: 1.42; 95% CI: 1.39–1.46), and were consistently lower in age groups older than 50 years.

**TABLE 3:** Herpes zoster incidence rates per 1,000 person‐years (95% CI), stratified by AD exposure status and age group.

	Incidence rate *per 1,000 person‐years*	
Age group (years)	*Exposed*	*Unexposed*
< 30	2.12 (2.07–2.17)	1.45 (1.44–1.47)
30–40	2.55 (2.49–2.62)	1.64 (1.62–1.67)
40–50	3.04 (2.97–3.11)	2.10 (2.08–2.13)
50–60	5.28 (5.18–5.37)	4.00 (3.96–4.04)
60–70	7.72 (7.60–7.84)	6.01 (5.96–6.06)
70–80	8.92 (8.78–9.07)	7.20 (7.14–7.26)
> 80	10.52 (10.33–10.71)	8.45 (8.36–8.53)

### Sensitivity analyses

**FIGURE 2:**
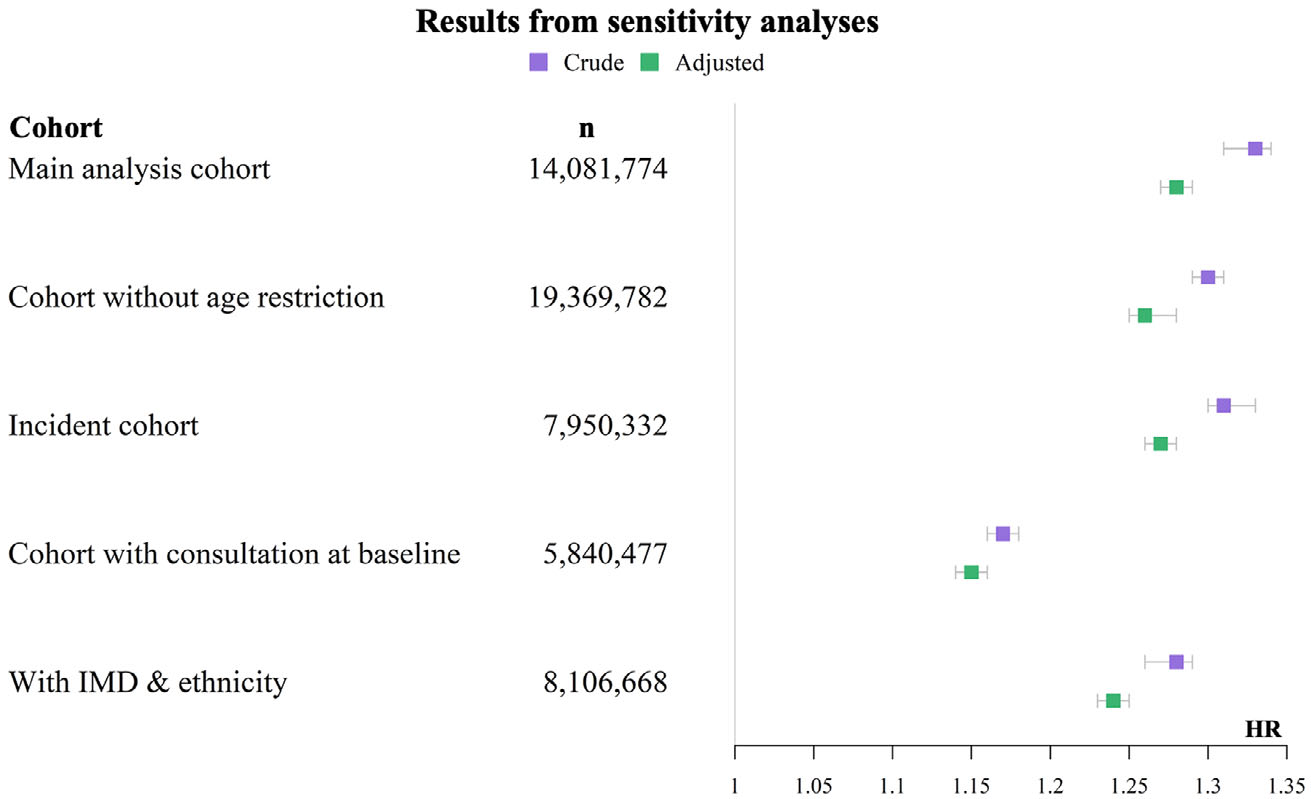
Forest plot summarizing the results of the sensitivity analyses, showing crude and adjusted hazard ratios (adjusted for age, sex, general practice, asthma, allergic rhinitis, COPD, coronary artery disease, hypertension, heart failure, ulcerative colitis, Crohn's disease, diabetes, depression, alcohol abuse, cigarette smoking, and obesity) and corresponding 95% confidence intervals for each analysis.

The results of the sensitivity analyses are summarized in Figure 2. Restricting the cohort to incident AD (1,382,577 observations with incident AD; aHR: 1.27; 95% CI: 1.26–1.28) and removing the age restriction (aHR: 1.26; 95% CI: 1.25–1.28) resulted in slightly lower but overall similar effect estimates in both the model accounting only for matching factors and the adjusted model compared with the main analysis (aHR: 1.28; 95% CI: 1.27–1.29).

Restricting the cohort to observations with any of the previously described common records on the index date or within the preceding year attenuated the effect estimates, reducing the aHR to 1.15 (95% CI: 1.14–1.16). Adding ethnicity and IMD to the adjusted Cox model in the main analysis cohort reduced the number of included observations to 8,106,668 and resulted in a slight attenuation of the effect estimates (fully adjusted HR: 1.24; 95% CI: 1.23–1.25).

## DISCUSSION

We found strong evidence of an association between AD and HZ, with an estimated 28% increased hazard of HZ (95% CI: 27%–29%) after adjustment for age, sex, calendar time, comorbidities, cigarette smoking, alcohol abuse, and obesity. The risk of HZ increased with increasing AD severity. Additional adjustment for exposure to oral corticosteroids and conventional immunosuppressants resulted in only a slight attenuation of the overall effect estimate. There was strong evidence of age‐related effect modification, with the effect of AD on HZ risk being more pronounced in younger age groups, particularly in individuals aged < 50 years.

Compared with previous studies investigating the association between AD and HZ, our study found a considerably lower excess risk than both a study using insurance data from Taiwan^20^ and a study based on US hospitalization data.^22^ Our results are consistent with those of a UK study by Wan et al.^19^ using a similar methodology, although that study investigated unspecified VZV infections in general and did not specifically focus on HZ. The study was based on UK primary care data from the THIN database, which is smaller overall and less representative than CPRD Aurum.^39^

Adjustment for different definitions of oral corticosteroid exposure and time‐updated immunosuppressant use resulted in only a slight attenuation of the effect estimates in the univariable model. While the increased risk of HZ associated with these treatments^40^ is reflected in the models, the findings suggest an elevated baseline risk of HZ in patients with AD, independent of comorbidities and conventional systemic treatment. Since the vast majority of observations were recorded before the introduction of JAK inhibitors into clinical practice at the end of 2022,^41^ the influence of these newer treatments on the results is likely negligible. At the same time, the findings may suggest that patients with AD are particularly vulnerable to HZ when treated with JAK inhibitors.

We observed a considerable increase in HZ risk with increasing AD severity, particularly among patients with severe AD. As treatments were used as a proxy for disease severity, disentangling the effects of increased severity from treatment‐related effects is challenging. Results from analyses exploring the mediating effects of oral corticosteroids, potent topical corticosteroids, and systemic immunosuppressants suggest partial mediation through these treatments.

Additional data from hospitals or specialist referrals, which were unfortunately not available in this study, would be needed to more precisely assess the effect of AD severity on HZ risk. The relationship is likely mediated by both the underlying disease severity and treatment‐specific immunomodulatory mechanisms.

The observed effect modification by age indicates that the effect of AD on HZ risk is stronger in younger adults than in older individuals. This may be related to factors influencing baseline risk that were not captured in the analysis and interact non‐multiplicatively with AD.

For instance, individuals aged > 70 years have been eligible for HZ vaccination since 2013,^42^ potentially attenuating hazard ratios in this age group, particularly if vaccination uptake differed between the exposed and unexposed groups. Additionally, as this study investigated only the first manifestation of HZ, survival bias may have played a role: if a relevant proportion of patients with AD experienced their first episode of HZ at a younger age, they would not have been included in the analysis of older age groups, potentially leading to an underestimation of age‐specific hazard ratios.

### Strengths and limitations

CPRD Aurum is broadly representative of the UK population with regard to geography, age, sex, and social deprivation. However, participation of GP practices in the database is voluntary, and patients may opt out, potentially affecting generalizability.

It is likely that AD is to some degree underdiagnosed or under‐documented.^43^ As AD status was recorded before the study outcome, any misclassification of exposure status can be assumed to be non‐differential, which would bias the effect estimates towards the null. Individuals not meeting the AD criteria were automatically classified as unexposed, which could introduce ascertainment bias.

Patients with AD are likely to consult their GP more frequently because of their disease. In addition, matched comparators may have included patients who did not consult their GP or did so only rarely, whereas exposed patients were required to have consulted their GP at least twice to meet the AD definition. Therefore, consultation bias may have inflated the effect estimates. Findings from the sensitivity analysis restricting the cohort to patients with at least one consultation in the year prior to baseline suggest some overestimation in the main analysis.

Data on comorbidities were extracted from medical records and may not have been fully captured because of underdiagnosis. In addition, treatment prescriptions were obtained only from primary care records, and CPRD does not capture adherence to prescribed treatments, limiting the sensitivity and specificity of these variable definitions.

As discussed before, the distinction between the effects of AD severity and treatments is limited by our methodology and should be interpreted with caution. Future research might consider alternative, score‐based classifications of disease severity, where available, to better disentangle these effects.

Residual confounding by targeted AD therapies or vaccination status remains possible and should be addressed in future research. However, unless vaccination uptake differed between the exposure groups, it is unlikely to have substantially influenced our estimates.

The study employed a dynamic cohort design including both prevalent and incident cases of AD to maximize sample size and enable the investigation of long‐term effects.^2^5 Sensitivity analyses restricted to incident AD cases yielded results similar to those of the main analysis. Reverse causality is unlikely, as the study was based on prospectively collected data and patients with an HZ diagnosis prior to the index date were excluded from the analysis. Due to the lack of documentation regarding reasons for censoring, the impact of loss to follow‐up could not be adequately assessed.

## Conclusions

Overall, these findings, considered in the context of the existing literature, suggest an elevated baseline risk of HZ in patients with AD, independent of comorbidities and conventional systemic treatment. This effect may be more pronounced in younger age groups. With emerging treatment options and broader implementation of HZ vaccination programs, incorporation of the evidence from this study into clinical practice and vaccination guidelines could improve the standard of care for patients with AD and help prevent HZ. To further improve our understanding of this association, additional research into the underlying biological mechanisms, as well as studies incorporating data on zoster recurrence, vaccination status, and a broader range of treatments, is warranted.

## CONFLICT OF INTEREST STATEMENT

None.

## Supporting information



Supporting Information
