# Supplementary material for: Atopic dermatitis is associated with herpes zoster in adults – A matched cohort study using electronic health records data in England
Source: J Dtsch Dermatol Ges. 2026 Aug 5;24(9):e1282–9. doi: 10.1111/ddg.70329 (PMC13551188; doi:10.1111/ddg.70329)
Supplement: Supplementary file 1 [file DDG-24-e1282-s001.pdf]

MedCode ID	Observations	Read Code (Original)	Read Code (Cleansed)	Term	Snomedct Concept ID
4765019	60000	A5315-1	A531511	Postherpetic neuralgia	2177002
8902018	700000	A53	A53..00	Herpes zoster infection	4740000
8904017	1000000	A53-1	A53..11	Shingles	4740000
18244018	100	A530	A530.00	Herpes zoster with meningitis	10491005
18585010	300	A5322	A532200	Herpes zoster iridocyclitis	10698009
30355011	8000	F300	F300.00	Post-herpetic trigeminal neuralgia	17974002
36819018	2000	A5311	A531100	Geniculate herpes zoster	21954000
145090014	30000	A5324	A532400	Herpes zoster ophthalmicus	87513003
286714015	30000	A532	A532.00	Herpes zoster with ophthalmic complication	186524006
286715019	1000	A5320	A532000	Herpes zoster with dermatitis of eyelid	186525007
286717010	1000	A532z	A532z00	Herpes zoster with other ophthalmic complication	186524006
286718017	2000	A53x	A53x.00	Herpes zoster with complication	49183009
286721015	500	A53xz	A53xz00	Herpes zoster with other specified complication NOS	49183009
286722010	700	A53y	A53y.00	Herpes zoster with unspecified complications	49183009
286723017	200000	A53z	A53z.00	Herpes zoster	4740000
288153012	50	AyuA4	AyuA400	[X]Zoster with other complications	4740000
288154018	100	AyuA5	AyuA500	[X]Zoster without complications	4740000
297557016	100	F3744	F374400	Polyneuropathy in herpes zoster	193190005
344957015	300	A5314	A531400	Zoster encephalitis	230176008
344958013	300	F0309-1	F030911	Herpes zoster encephalitis	230176008
395349015	20000	A531	A531.00	Herpes zoster with nervous system complication	111861003
481174015	1000	A53x0	A53x000	Herpes zoster otitis externa	21954000
1219080012	40	F0112-1	F011211	Herpes zoster meningitis	10491005
1229869010	2000	A5321	A532100	Herpes zoster with keratoconjunctivitis	42448002
4.41E+13	10000	A5323	A532300	Ophthalmic herpes zoster infection	87513003
1.92E+14	10000	A5311-1	A531111	Ramsay Hunt syndrome	21954000
2.11E+14	1000	A5313	A531300	Post-herpetic polyneuropathy	76462000
2.11E+14	5000	A5312	A531200	Postherpetic trigeminal neuralgia	17974002
2.13E+14	20000	A5315	A531500	Post-zoster neuralgia	2177002

MedCode ID	Observations	Read Code (Original)	Read Code (Cleansed)	Term	Snomedct Concept ID
2.17E+14	100000	A531-1	A531.11	Post-herpetic neuralgia	2177002
4.87E+14	7000	65O2	65O2.00	Anti-varic-Zoster immunoglob.	373873005
6.25E+14	100	A53x1	A53x100	Disseminated herpes zoster	55560002
6.41E+14	100	F0309	F030900	Encephalitis due to herpes zoster	230176008
7.06E+14	100	F0112	F011200	Herpes zoster with meningitis	10491005
7.79E+14	300	F5016	F501600	Infective otitis externa due to herpes zoster	21954000
8.22E+14	100	F5016-1	F501611	Herpes zoster - otitis externa	21954000
8.22E+14	200	A531z	A531z00	Herpes zoster with other CNS complication NOS	111861003
8.22E+14	400	A5310	A531000	Herpes zoster with other CNS complications	111861003
8.79E+14	4000	A532-99	A532.99	Ophthalmic herpes zoster	186524006
8.79E+14	1000	A53z-99	A53z.99	Other herpes zoster	6.65E+14
9.06E+14	5000	HNG0041		[RFC] Shingles	9.06E+14
9.06E+14	900	HNG0060		[RFC] Shingles	9.06E+14
9.25E+14	1	PCNQAN6		Anti-varic-zoster immunoglob.	9.25E+14
9.89E+14	20	A5323-99	A532399	Ophthalmic herpes zoster	87513003
1.13922E+15	50	2Fa	2Fa..00	Hutchinson's sign - herpes zoster involving nose tip	410500004
2.53365E+15	500	^ESCTPH253365		PHN - Post-herpetic neuralgia	2177002
2.57447E+15	1	^ESCTZO257447		Zoster	4740000
2.66688E+15	6	^ESCTVA266688		Varicella-zoster meningitis	10491005
2.81012E+15	4	^ESCTVA281012		Varicella-zoster virus	19551004
2.81014E+15	6	^ESCTVZ281014		VZV - Varicella-zoster virus	19551004
2.81016E+15	1	^ESCTHZ281016		HZ - Herpes zoster virus	19551004
2.81017E+15	2	^ESCTHE281017		Herpes zoster	19551004
2.84916E+15	100	^ESCTHE284916		Herpes zoster auricularis	21954000
2.84919E+15	30	^ESCTHE284919		Herpes zoster oticus	21954000
2.84921E+15	30	^ESCTHZ284921		HZO - Herpes zoster oticus	21954000
2.84924E+15	1000	^ESCTRA284924		Ramsay Hunt syndrome - auricular	21954000
2.84926E+15	3	^ESCTRA284926		Ramsay Hunt syndrome 2	21954000
3.1606E+15	10	^ESCTRA316060		Ramsay Hunt syndrome 1	41009006

MedCode ID	Observations	Read Code (Original)	Read Code (Cleansed)	Term	Snomedct Concept ID
3.18E+15	30	^ESCTHE318441		Herpes zoster keratoconjunctivitis	42448002
3.74382E+15	3	^ESCTNE374382		Nerve pain from shingles	76462000
3.92247E+15	2	^ESCTZO392247		Zoster ophthalmicus	87513003
4.74225E+15	10	^ESCTHE474225		Herpes zoster dermatitis of eyelids	186525007
4.76906E+15	20	^ESCTRA476906		Ramsay Hunt syndrome - ataxia	192871008
5.0092E+15	20	^ESCTAC500920		Acute trigeminal herpes zoster	230536009
5.00987E+15	50	^ESCTIN500987		Intercostal post-herpetic neuralgia	230597003
5.07455E+15	6	^ESCTHE507455		Herpes zoster infection of oral mucosa	235059009
5.12462E+15	1	^ESCTZO512462		Zosteriform lichen planus	238648000
5.14817E+15	200	^ESCTTH514817		Thoracic herpes zoster infection	240470005
5.14818E+15	50	^ESCTLU514818		Lumbar herpes zoster infection	240471009
5.14819E+15	9	^ESCTMA514819		Maxillary herpes zoster infection	240472002
5.1482E+15	3	^ESCTMA514820		Mandibular herpes zoster infection	240473007
5.14821E+15	3	^ESCTMU514821		Multidermatomal herpes zoster infection	240474001
5.41961E+15	20	^ESCTVA541961		Varicella-zoster virus antibody	259858000
5.98924E+15	1000	^ESCTVA598924		Varicella-zoster virus infection	309465005
6.58913E+15	400	^ESCTHE658913		Herpes zoster keratitis	397573005
6.90E+15	1	^ESCTVA689615		Varicella-zoster virus eyelid dermatitis	416718008
6.96478E+15	1	^ESCTHE696478		Herpes zoster retinitis	421029004
6.99047E+15	1	^ESCTHE699047		Herpes zoster acute retinal necrosis	422471006
7.02471E+15	4	^ESCTCO702471		Corneal haze due to herpes zoster	424353002
7.03323E+15	60	^ESCTHE703323		Herpes zoster corneal endotheliitis	424801004
7.03325E+15	3	^ESCTHE703325		Herpes zoster corneal endothelialitis	424801004
7.57633E+15	1	^ESCTVA757633		Varicella zoster virus IgG antibody	703486008
9.87162E+15	1	^ESCTKE987162		Keratitis of right eye due to herpes zoster	1.60E+16
1.37148E+16	1	^ESCT1371484		Herpes zoster conjunctivitis of right eye	1.59893E+16
1.40128E+16	1	^ESCT1401284		Bilateral herpes zoster keratitis	1.59924E+16
